# Graphene‐Based Polymer Bilayers with Superior Light‐Driven Properties for Remote Construction of 3D Structures

**DOI:** 10.1002/advs.201600437

**Published:** 2017-01-24

**Authors:** Zhenhua Tang, Ziwei Gao, Shuhai Jia, Fei Wang, Yonglin Wang

**Affiliations:** ^1^ School of Mechanical EngineeringXi'an Jiaotong UniversityXi'an710049China

**Keywords:** 3D assembly, bilayer polymeric composite, graphene, light‐driven

## Abstract

3D structure assembly in advanced functional materials is important for many areas of technology. Here, a new strategy exploits IR light‐driven bilayer polymeric composites for autonomic origami assembly of 3D structures. The bilayer sheet comprises a passive layer of poly(dimethylsiloxane) (PDMS) and an active layer comprising reduced graphene oxides (RGOs), thermally expanding microspheres (TEMs), and PDMS. The corresponding fabrication method is versatile and simple. Owing to the large volume expansion of the TEMs, the two layers exhibit large differences in their coefficients of thermal expansion. The RGO‐TEM‐PDMS/PDMS bilayers can deflect toward the PDMS side upon IR irradiation via the cooperative effect of the photothermal effect of the RGOs and the expansion of the TEMs, and exhibit excellent light‐driven, a large bending deformation, and rapid responsive properties. The proposed RGO‐TEM‐PDMS/PDMS composites with excellent light‐driven bending properties are demonstrated as active hinges for building 3D geometries such as bidirectionally folded columns, boxes, pyramids, and cars. The folding angle (ranging from 0° to 180°) is well‐controlled by tuning the active hinge length. Furthermore, the folded 3D architectures can permanently preserve the deformed shape without energy supply. The presented approach has potential in biomedical devices, aerospace applications, microfluidic devices, and 4D printing.

## 1 Introduction

Employing 2D planar polymer sheets to fabricate complex 3D structures has been the subject of much research in recent years, and has several attractions and technological advantages.^1^ First, 2D polymer sheets can be mass‐produced inexpensively. Second, planar sheets can be stacked efficiently for storage, transport, or remote deployment. Finally, planar materials are compatible with a wide range of fabrication techniques. Because of these properties, 2D planar polymer sheets have proven to be promising in the design of flexible biomedical devices,^2^ 3D cell‐laden microstructures,^3^ self‐assembling microfluidics,^4^ aerospace structural components,^5^ deformable batteries,^6^ and soft robot fabrication.^7^

In general, there are two main strategies for triggering an out‐of‐plane shape change in 2D planar polymers to construct 3D structures: bending and folding. Folding is actually localized bending, so these terms are sometimes used interchangeably.^8^ So far, many stimuli‐responsive polymers have been investigated and employed to fabricate 3D structures including hydrogels,^9^ shape memory polymers,^10^ thermoresponsive polymers,^11^ and gradient polymeric composites.^12^ Moreover, the frequently used external stimuli to trigger a shape change in stimuli‐responsive polymers include solvents,^13^ electricity,^14^ pneumatic stimulus,^15^ mechanical stimulus,^16^ heat,^17^ and light.^18^ Electrical stimuli allow sequential folding with high accuracy, but the structures must be wired to external controls. Although pneumatics can also induce out‐of‐plane motion from a 2D substrate, this method requires the inconvenience of tethering inlets and outlets for the pneumatic gas. Mechanical stimuli also can realize autonomic assembly of 3D structures across a wide range of length scales and material types. However, the delamination and fracture from the defects in the fabrication process may appear during the assembly. Temperature‐responsive composites (e.g., poly(*N*‐isopropylacrylamide)[[qv: 17c]] and hydrogels^11^) have been widely used as precursor materials in 3D structures. However, these deformation processes require extra solvent media and are generally slow owing to the reliance on the diffusion of the solvent into the polymer.^13^ Among the above‐mentioned external stimuli, light stimulus is particularly attractive owing to its distinct advantages over other triggers, such as its remote and wireless characteristics and its spatial and temporal controllability. To date, light‐activated polymer sheets have been explored to fabricate 3D macro‐ or microscopic architectures, such as the use of photoinduced stress relaxation of polymers to fabricate 3D configurations.^19^ Another common approach is to dope stimuli‐responsive materials with photothermal materials or other photosensitive molecules to take advantage of light‐triggered swelling.[[qv: 17d,18b,c]] However, the fabrication processes of these precursor sheet materials are complicated, and sometimes the extra mechanical stimuli (prestrain) requires physical contact with the precursor samples to program the material with the information required to enable it to subsequently fold itself.^8^ These drawbacks severely limit the practical applications of light‐activated sheets. In summary, all of the aforementioned approaches can effectively realize 3D fabrication, but these strategies cannot easily construct complex 3D configurations or the fabrication process, actuation type, and working condition still require improvement. Hence, the development of light stimulus and easily prepared smart polymer sheets with a large deformation ability, high stability, and a high mechanical property to fabricate 3D complex structures is of great interest and significant importance, and still remains a major challenge.

Graphene,^20^ because of its miraculous properties of high electrical and thermal conductivity and high mechanical performance has been widely used as a filler material to boost the overall performance of graphene‐based polymer composites.^21^ Furthermore, graphene, which also possesses a high photothermal conversion efficiency,^22^ inspired us with a feasible way to realize photomechanical actuators with excellent performance.^23^ Herein, we present a feasible and scalable method for the fabrication of light‐driven polymeric composites based on reduced graphene oxides (RGOs) and thermally expanding microspheres (TEMs) that takes advantage of these materials' highly intriguing photothermal properties and large volume expansivity, respectively. The use of RGOs and TEMs in the polymer templates yields an excellent route toward IR optically responsive polymer materials. Our light‐driven composites consist of two layers with different properties, where the active layer is an RGO‐TEM‐PDMS (poly(dimethylsiloxane)) hybrid composited layer and the passive layer is pure PDMS. Each layer of the RGO‐TEM‐PDMS/PDMS composite has a different coefficient of thermal expansion (CTE) and Young's modulus owing to the existence of the RGOs and TEMs. The polymeric bilayers are simply achieved using tape casting processing, and exhibit significant photo‐thermoinduced bending deformation behaviors upon IR irradiation owing to the photothermal effect of the RGOs and the remarkable expansion of the TEMs. Specifically, these RGO‐TEM‐PDMS/PDMS bimorph composites with large bending deformation and favorable stability are used as active hinges to build 3D structures such as bidirectionally folded columns, cubic boxes, pyramids, and cars. The extent and folding direction of the hinge bending or folding was controlled by varying the length of the active hinge and changing the hinge layout, respectively. This investigation provides an effective and versatile method for fabricating 3D configurations from programmable 2D precursors and provides insight into how the relevant mechanisms of photo‐thermomechanical conversion affect the deformation properties of the proposed bilayer systems.

## 2 Results and Discussion

### 2.1 Design and Fabrication of Light‐Driven Polymeric Bilayers with Excellent Bending Properties

**Figure 1:**
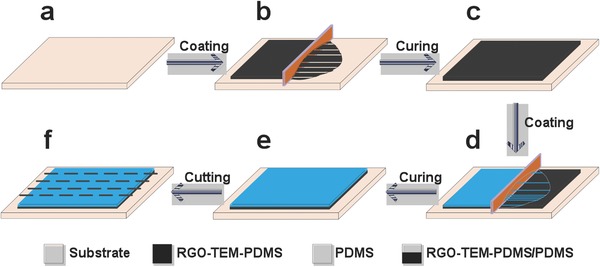
Schematic of the fabrication procedure for the infrared‐triggered bilayer configuration. a) A clean glass substrate. b) The bottom layer RGO‐TEM‐PDMS solution is cast on the substrate. c) The bottom layer is cured to form a thin RGO‐TEM‐PDMS membrane with a relatively uniform thickness. d) A layer of PDMS solution is coated on the bottom layer to obtain a bimorph structure. e) The layers are cured again to form an RGO‐TEM‐PDMS/PDMS bilayer. f) The bilayer is cut into 2D rectangular strips along the dashed lines.

In this study, the photo‐thermoinduced bending characteristics of the RGO‐TEM‐PDMS/PDMS bilayer actuator were achieved by designing an asymmetrical distribution of fillers across the thickness. When the IR light was absorbed by the RGOs, the polymeric bimorph experienced a temperature elevation and deflected to the PDMS side owing to the different thermal expansions of each layer. We selected PDMS elastomer as the passive substrate and the host matrix because of its optical transparency, nontoxicity, remarkable durability against repeated deformation, and resistance to high temperature. Because thermally RGO has a comparatively higher IR absorption characteristic than graphene, we employed RGOs to work as the energy transfer units.[[qv: 22b]] In addition, TEMs were selected as the mechanical deformation units owing to their large volume expansion upon heating. **Figure** **1** schematically shows the detailed fabrication process of the RGO‐TEM‐PDMS/PDMS bilayer film. The process is facile and scalable, and only involves tape‐coating processes for both the bottom and top layers. First, to prepare the bottom RGO‐TEM‐PDMS film, appropriate amount of RGOs, TEMs, and PDMS (ratio of base to cross‐linker 10:1 by mass) was mixed into a solution by a sonication dispersion method. Then, the mixture solution was coated onto a glass substrate by a tape casting coater, and the formed thickness was adjustable (0–5000 µm thickness using a micrometer‐adjustable applicator). Second, the formed layer was cured in an oven at 60 °C for 1 h to fabricate the incomplete cured bottom RGO‐TEM‐PDMS layer with a thickness of ≈0.7 mm. Finally, the preprepared viscous PDMS was poured on the RGO‐TEM‐PDMS layer with a coating process by the tape casting coater and fully cured at 60 °C for 4 h in an oven to fabricate the final RGO‐TEM‐PDMS/PDMS bilayer structure. The total thickness of the bilayer was ≈1.4 mm, and polymeric bilayer strips were cut for subsequent experimental measurements. Details of the fabrication process are given in the Experimental Section (Section 4). Unless otherwise specified, the concentration of RGO and TEM are 0.3 and 30 wt%, respectively.

### 2.2 Morphological Analyses of RGO‐TEM‐PDMS/PDMS Bilayer Films

**Figure 2:**
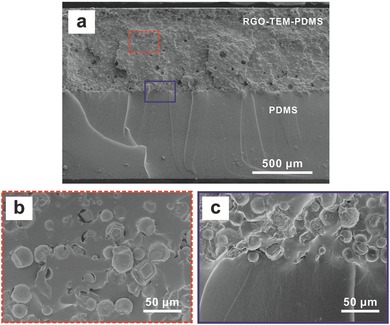
Morphology characterization. a) The cross‐sectional FE‐SEM image of the RGO‐TEM‐PDMS/PDMS bilayer composite with 0.3 wt% RGO and 30 wt% TEM. The PDMS layer (bottom) is smooth. b,c) Magnified FE‐SEM images of the areas in (a) showing the (b) RGO‐TEM‐PDMS layer (red dashed square) and the (c) bilayer interface (purple solid square).

To characterize the RGO‐TEM‐PDMS/PDMS bilayers, their initial internal morphology was observed with a field emission scanning electron microscope (FE‐SEM). **Figure** **2**a shows the cross‐sectional FE‐SEM image of the RGO‐TEM‐PDMS/PDMS film, where the thickness of the bimorph structure is ≈1.4 mm in total, the RGO‐TEM‐PDMS layer (top) is ≈0.7 mm thick and the PDMS layer (bottom) is ≈0.7 mm thick. From this figure, it can be noted that tight RGO‐TEM‐PDMS/PDMS hybrid bilayers with a distinct layer structure are obtained by our present approach. Figure 2b shows a higher magnification FE‐SEM image of the TEMs in the RGO‐TEM‐PDMS layer, which exhibit a uniform distribution. The uniformity of the microspheres in the bilayer structure has a remarkable influence on their actuation performance. Moreover, the image in Figure 2b also shows no indication of large agglomerations or clustering of the RGOs and TEMs, thereby signifying a uniform dispersion of RGOs and TEMs in the PDMS host matrix. A truly homogeneous dispersion of graphene materials in the host polymer matrix directly correlates with the effectiveness of the composite for IR absorption. As shown in the higher‐magnification FE‐SEM image of the interface (Figure 2c), plenty of cross‐links are formed across the interface owing to the interactions of the polymer chains, which locks the two layers of the RGO‐TEM‐PDMS/PDMS together tightly.

### 2.3 Properties of Polymer Composite Films

**Figure 3:**
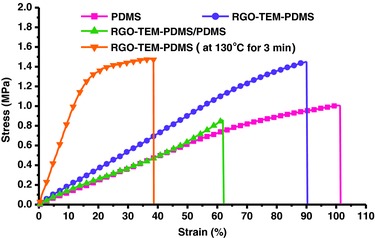
Stress–strain curves of pure PDMS, RGO‐TEM‐PDMS, and RGO‐TEM‐PDMS/PDMS composite films at room temperature.

The mechanical properties of pure PDMS, RGO‐TEM‐PDMS, and RGO‐TEM‐PDMS/PDMS are shown in **Figure** **3** and Table S1 (Supporting Information). The RGO‐TEM‐PDMS at 0.3 wt% RGO and 30 wt% TEM showed a Young's modulus of 1.82 MPa, whereas the modulus of pure PDMS was 1.21 MPa. The difference in Young's modulus determines the bending properties of the RGO‐TEM‐PDMS/PDMS bilayer platform. What is more, the maximum CTE of RGO‐TEM‐PDMS was about 2 × 10^−3^ K^−1^, six times higher than the CTE of the pure PDMS (less than 0.31 × 10^−3^ K^−1^). The two layers with large different CTEs possess different IR‐induced expansion rate and different IR‐induced mechanical forces. As a result, the RGO‐TEM‐PDMS/PDMS bilayers exhibit significant photo‐thermoinduced bending properties due to their large differences in CTE and modulus. Interestingly, the tensile strength of the expanded RGO‐TEM‐PDMS films (after heating of specimens at 130 °C for 3 min) is about 1.47 MPa, which is little higher than that of the original RGO‐TEM‐PDMS films (1.45 MPa). Moreover, the Young's modulus is increased by 470% from 1.82 MPa for original RGO‐TEM‐PDMS film to 8.61 MPa for expanded RGO‐TEM‐PDMS film (see Table S1 in the Supporting Information). These results indicated that the deformed RGO‐TEM‐PDMS/PDMS composites become stronger and stiffer, thus making these composites highly promising in the realm of functional materials. It is well‐known that the agglomeration and poor dispersion of fillers in composites cause crack initiation and propagation, which would result in decrease of tensile strength for the materials. Thus, the observed increased tensile strength of RGO‐TEM‐PDMS (compared with PDMS, see Table S1 in the Supporting Information) could be considered as indirect evidence of the homogeneous dispersion of fillers in the PDMS matrix.

### 2.4 Photothermal Deflection of RGO‐TEM‐PDMS/PDMS Bilayer Films

Because of the existence of TEMs and RGOs in our system, the composited RGO‐TEM‐PDMS layer possessed a dramatically different CTE and Young's modulus than that of the pristine PDMS layer, and these differences in CTEs and Young's moduli determine the photothermal properties of the RGO‐TEM‐PDMS/PDMS bilayers. The TEMs have a large volume expansion of ≈4000%, and the incorporation of fillers with a large CTE value would have a positive effect on the CTE value of the composite. Given the constituents of the RGO‐TEM‐PDMS layer in our bimorph structures, it is reasonable that the CTE of this layer will increase owing to the existence of the TEMs. This CTE increase will cause a deflection toward PDMS layer just as the common phenomenon in bilayer thermal structures. Furthermore, the static performance of the bilayer sheet was modeled using relevant expressions from linear beam theory (see the Supporting Information for details).

**Figure 4:**
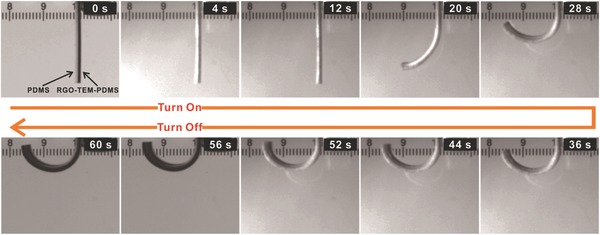
Optical photographs of the dynamic photothermal deflection of an RGO‐TEM‐PDMS/PDMS bilayer. The IR light source is positioned 40 mm from PDMS side (left side of the image). The bimorph bends to a complete semicircle under the light irradiation with a deformation angle of 180°, and the final shape retains this transformed state when the light is off.

To investigate the photothermal behavior of these RGO‐TEM‐PDMS/PDMS bilayer films induced by IR irradiation, the sample strips with dimensions of 20 × 2 × 1.4 mm^3^ and containing 0.3 wt% RGO and 30 wt% TEM were exposed to the IR lamp (Philips BR125), where the IR light source was positioned 40 mm from the PDMS side (see Video S1 in the Supporting Information). Photographs of the dynamic photothermal deflection of the RGO‐TEM‐PDMS/PDMS bilayer are shown in **Figure** **4**. From this figure, it can be seen that the bilayer structure bends toward the PDMS side when IR light is irradiated on the PDMS side, until the structure becomes almost semicircular (180°) within ≈30 s. When the IR light is then turned off, the bilayer structure retains its bent shape permanently, which indicates the excellent durable retention behavior (long‐time service) of these bilayer films with no energy supplied. Moreover, the strength and stability of the ultimate bent shape structures were quantitatively confirmed by experiments. The unbending force (to drive the ultimate C‐shaped structure to its initial straight state) was measured by using a microforce testing system (Tytron 250, USA). Approximately 110 mN (maximum) was required to drive the C‐shaped structure to its initial straight state. A noteworthy point is that the permanent bending structure can bear 140 times (6.16 g/0.043 g) its own weight. This behavior is expected because the stiffness of the bending beam increases due to the plastic deformation of the microspheres, which allows the bending beam to generate a larger force. These results indicate that the proposed material approach can be implemented for the development of high‐performance foldable electronic devices and soft robotics bodies.

The bending deformation of the RGO‐TEM‐PDMS/PDMS bilayer strips results from the large differences in the CTEs existing between the RGO‐TEM‐PDMS and PDMS layers (see Table S1 in the Supporting Information). Upon exposure to IR irradiation, light is absorbed by the RGOs within the PDMS matrix and the optical energy is efficiently transduced into thermal energy through phonons in the sp^2^ graphene sheets.^22^ Owing to the high thermal conductivity of graphene films and the intimate dispersion of RGOs within the PDMS matrix, heat is percolated through the matrix. This thereby causes the TEMs contained in the layer to increase in temperature. The large difference in the CTEs leads to an asymmetric expansion in the RGO‐TEM‐PDMS and PDMS layers when the temperature changes, and an interfacial stress is therefore produced to roll the RGO‐TEM‐PDMS/PDMS bilayer films. Here, the absorption of the optical energy takes place by RGOs, which transfers the absorption energy to the TEMs (mechanical deformation unit) whereupon a volume expansion takes place. According to the intrinsic properties of the TEMs, the microspheres do not shrink after the IR light is turned off. Therefore, the RGO‐TEM‐PDMS/PDMS bilayers bent to the PDMS side under IR stimulus permanently. For the RGO‐TEM‐PDMS/PDMS strip, the bending direction of the bimorph layer induced by IR irradiation incident on either side of the layer was the same (see Videos S1 and S2 in the Supporting Information). The RGO‐TEM‐PDMS side with a larger expansion ratio was always convex, while the plain PDMS side with the smaller expansion ratio was always concave.

**Figure 5:**
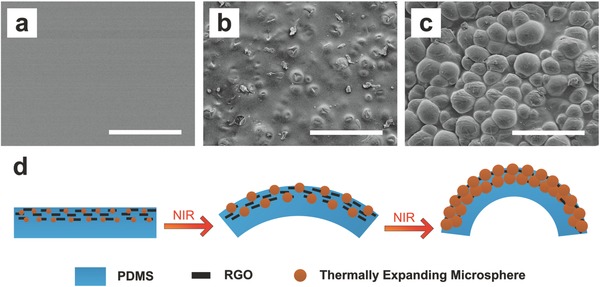
Effects of thermally expanding microsphere expansion. a) FE‐SEM image showing the smooth surface of the as‐fabricated composites (RGO‐TEM‐PDMS layer side, 0.3 wt% RGO, 30 wt% TEM). b) FE‐SEM of the same composite sample after slight expansion showing a bumpy surface. c) FE‐SEM of the same composite sample postexpansion with microspheres clearly visible. d) Schematic of the bending mechanism of the bilayer structure. All scale bars represent 200 µm.

To better understand the photothermal mechanism under IR illumination, the surface micromorphologies of a series of bilayers at three different deformation stages (Figure S1, Supporting Information) were observed by FE‐SEM. **Figure** **5**a shows the smooth surface (RGO‐TEM‐PDMS side) of an as‐fabricated unexpanded bilayer composite (0.3 wt% RGO, 30 wt% TEM). In this image, neither the RGOs nor TEMs are distinguishable from the bulk polymer matrix. After IR irradiation for 15 s, the bilayer sample exhibits a slight deflection (not yet attained maximum deformation) and the TEMs are seen bulging out of the bulk polymer (see Figure 5b). After the samples have reached the maximum bending state following 15 s of irradiation, postexpansion microspheres (see Figure 5c) are clearly seen bulging from the bulk polymer. The underlying bending mechanism is schematically depicted in Figure 5d. From Figure 5a–c, it can be observed that the TEMs of the bilayers at different swelling deformation stages present a different morphology. Namely, the original TEMs are small spherical plastic particles whose volume gradually increases when the temperature rises via IR irradiation. These results indicate that TEM expansion is the main effect contributing to the deformation mechanism. In order to gain more insight into the expansion mechanism of TEMs intuitively, FE‐SEM images of the microspheres at different temperature stages are shown in Figure S2 (Supporting Information). Using FE‐SEM, the initial microsphere diameter was determined to be 24.0 ± 3.2 µm, and expanded 86.5 ± 11.1 µm (yielding 350% diameter and 4000% volume increases).

**Figure 6:**
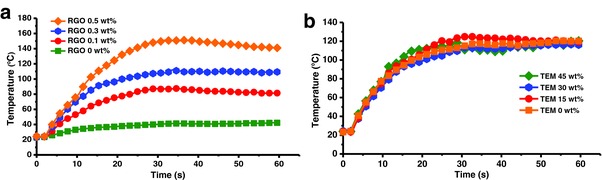
Corresponding temperature–time curve during the IR irradiation process. a) Time‐dependent surface temperature measurements of the polymeric bilayer sheets after irradiation initiation at 2 s for different RGO concentrations (TEM 30 wt%). b) Temperature profile of the polymeric bilayer sheets after irradiation initiation at 2 s for different TEM concentrations (RGO 0.3 wt%).

To reveal the IR absorption and photothermal conversion brought about by the RGOs, an IR camera was employed to record the real‐time temperature of the samples during the irradiation process. **Figure** **6**a plots the temperature profile at the irradiation area of various polymeric bilayer strips comprising 30 wt% TEM and different RGO concentrations ranging from 0 to 0.5 wt%. It can be seen that the temperature increases rapidly, except in the specimen without RGOs, and reaches a maximum at ≈30 s. The maximum temperature and the corresponding temperature response rate increases with the RGO concentration. However, under the same irradiation condition, no obvious temperature change for the TEM‐PDMS/PDMS bilayer (without RGOs) is observed (see Figure 6a). Therefore, it can be concluded that the RGOs act as the energy transfer units. The maximum temperature and the temperature response rate are highly dependent upon the RGO concentration, which is relevant to understand the enhancement of photothermal conversion and faster response times in specimens with higher RGO concentrations. This result agrees with a previous finding wherein RGOs tended to enhance the photothermal conversion.^23^ To further elucidate the role of the RGOs as energy transfer units separate from the TEMs, the temperature of a series of bilayer strips with different TEM concentrations ranging from 0 to 45 wt% was also measured. Figure 6b shows the surface temperature as a function of irradiation time for bilayer samples comprising 0.3 wt% RGO and different TEM concentrations. From this figure, it can be noted that the surface temperature is independent of the TEM content. That is to say, the TEMs do not essentially change the photothermal conversion properties of the polymeric composites owing to their poor IR absorption. The comparison experiment clearly confirms that RGOs are the energy transfer units that effectively absorb the IR wavelength and convert the light energy to thermal energy.

**Figure 7:**
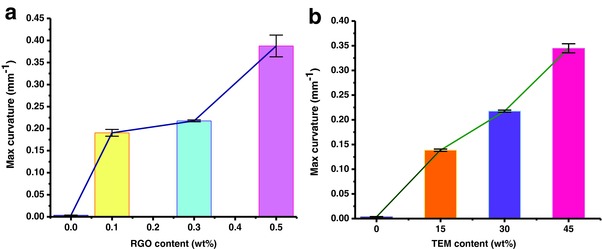
The maximum curvature of the RGO‐TEM‐PDMS/PDMS bilayer with different filler loading in active layer. a) The maximum curvature change with different RGOs content (TEM 30 wt%). b) The maximum curvature change with different TEMs content (RGO 0.3 wt%). Four samples were tested for each film type.

For characterization, the photomechanical properties of bilayer structures with different filler (RGOs or TEMs) concentrations were tested using beam‐shaped structures with dimensions of 20 × 2 × 1.4 mm^3^. **Figure** **7** shows the dependence of the maximum curvature on the filler loading. From Figure 7a, it can be noted that the curvature increases with the RGO concentration because the temperature increased with an increase of graphene content (see Figure 6a), which is the origin of the expansion of the TEMs, increases with the concentration of the RGO. In particular, the curvature increases significantly when RGO content is higher (0.5 wt%). The large curvature changes of the high content RGO incorporated bilayers are ascribed to the combined effects of the higher thermal conductivity of the active layer and the higher raised‐temperature. Hence, it is further confirmed that RGO is energy transfer unit. From Figure 7b, it can be noted the curvature typically increases with the TEM concentration, and ultimately reaches a value of about 0.35 mm^−1^ at 45 wt% TEM concentration. This confirms that the photomechanical deflection exhibits a positive correlation with the TEM concentration, and suggests that the TEMs are the principle factor for the occurrence of self‐bending (i.e., TEMs act as the mechanical deformation units). While a high wt% of TEM is desirable from an expansion perspective, there are associated drawbacks. Increasing the TEM loading may result in structures that are unstable and that tend to break apart or crack upon expansion.

**Figure 8:**
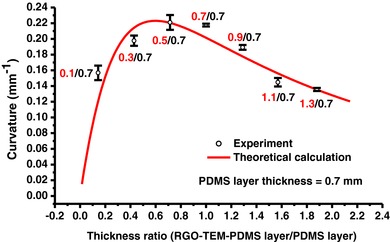
The experimental (black symbols) and theoretical (red lines) curvature as a function of the thickness ratio (RGO‐TEM‐PDMS layer/PDMS layer thickness) for RGO‐TEM‐PDMS/PDMS bilayers with different RGO‐TEM‐PDMS thicknesses. The model parameters for these computations were obtained from experimental data (Table S1, Supporting Information).

In this study, another consideration in the design is the relative thickness of the bilayer sheet. Effects of the thickness ratio (RGO‐TEM‐PDMS layer to PDMS layer) on the curvature of the bilayer sheets are presented in **Figure** **8**. Here, we varied the thickness of RGO‐TEM‐PDMS layer (active layer) and maintained the PDMS layer (passive layer) thickness to be constant at ≈0.7 mm. From this figure, it can be noted that the curvature reaches its maximum point when the thickness ratio is around 0.7 and then decreases again afterward. The theoretical curvatures are in good agreement with the experiments. According to this figure, the active layer with moderate thickness leads to higher bending curvature, therefore, using an active layer thickness of ≈0.7 mm for experiments is beneficial, and it is easy to fabricate and offers a large curvature.

Moreover, as another important performance indicator, the output force of the RGO‐TEM‐PDMS/PDMS bilayers during the deformation process was investigated (see Figure S3 in the Supporting Information). The graph shows the vertical blocking force increased with irradiation time, and the maximum output force is found to be ≈5.5 mN after 50 s irradiation, followed by a slow increase. This slow increase of the output force of the bilayer is mainly attributed to the CTE and the temperature gradient (across the composite beam) decreased when the temperature was raised above 100 °C. Blocking force divided by the weight of an actuator (the force density) is a measure of the effective force generation of an actuator. A bilayer system requires the force density over 1 for an effective bending motion.^24^ In our system, the force density was over 13 (the bilayer weight was 41 mg), showing powerful actuation of the graphene‐based bilayer structures. Since the RGO‐TEM‐PDMS/PDMS bilayers are lightweight and the exerted force of the bilayers is large, a number of applications (such as active origami, soft robots, and smart shape control devices) may be developed.

### 2.5 3D Configurations Constructed from 2D Structures

**Figure 9:**
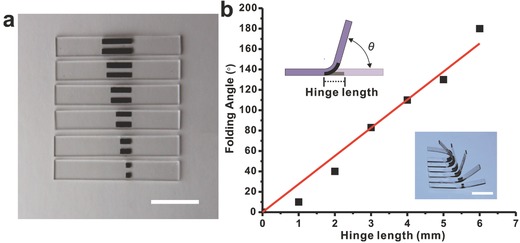
Self‐folding deformation of the hinge actuator when is exposed to light. a) Series of hinge actuators (25 × 4 × 1.4 mm^3^) with hinge lengths ranging from 1 to 6 mm (from bottom to top). b) Measured (black symbols) and fitted (red lines) maximum folding angle as a function of the hinge length. These samples are exposed to a 250 W IR light source. (Insets) Schematic (upper) and corresponding maximum deformed shapes (lower). All scale bars represent 10 mm.

The outstanding light‐driven property of these RGO‐TEM‐PDMS/PDMS bilayer films when used as active hinges makes it a promising material to construct complex 3D configurations from corresponding 2D bilayers. In this study, we deformed the polymer sheet by bending and folding. Folding is simply a bend that is localized within a narrow, rectangular region that we call a hinge, which results in the plates on each side rotating at an angle with respect to each other. To obtain actuation hinges of different sizes, RGO‐TEM‐PDMS/PDMS bilayer strips with different lengths (0.7/0.7 mm thickness and 1 mm width unless otherwise noted) were directly connected to the body substrates by the cross‐linking of the matrix and used as intelligent active hinges to create origami folding patterns (more details of the fabrication process are described in Figure S4 in the Supporting Information). Here, a series of simple active hinge configurations were designed and fabricated, which consisted of two RGO‐TEM‐PDMS/PDMS bilayer strips placed horizontal to each other and two pristine PDMS plates attached onto the opposite ends of the bilayer strips as panels, as shown in **Figure** **9**a. These original hinge actuators with identical dimensions (25 × 4 × 1.4 mm^3^) and varying active bilayer hinge lengths (*l* = 1–6 mm, from bottom to top of Figure 9a) were exposed to external IR irradiation stimuli at a set distance of 40 mm from the IR lamp. The folding angle is defined as the rotation angle of a folded plane (Figure 9b, upper inset). Figure 9b plots the maximum fold angle where the TEMs are fully expanded as a function of the hinge length. It can be noted that the hinge angle increases with the hinge length, and the experimental data can be accurately described by a linear relationship across the full range of 0°–180° (red line in Figure 9b). Therefore, we can precisely control the hinge angle in a range from 0° to nearly 180° simply by varying the active hinge length. These results are subsequently used to choose the appropriate active hinge length to induce a desired fold angle in a hinge structure.

**Figure 10:**
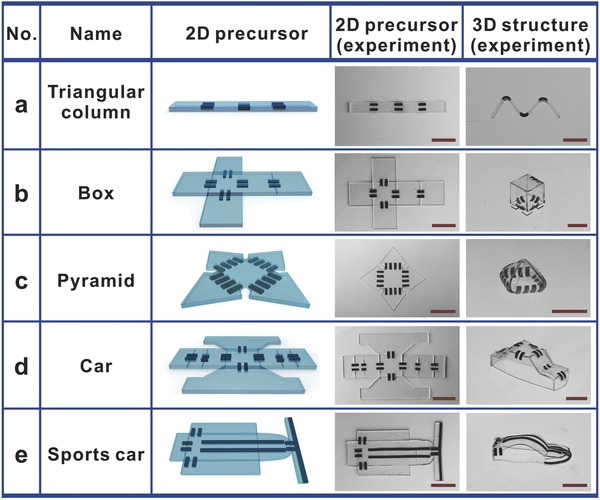
Representative examples of the origami assembly of 3D structures from corresponding 2D sheets. a) 2D precursors and experimental (optical) images of triangular columns. b–f) Similar results that describe the formation of (b) a box, (c) a pyramid, (d) a car, and (e) a sports car. All scale bars represent 10 mm.

A diverse range of basic origami‐inspired geometries can be realized with these simple approaches. We created a number of examples that demonstrate prefabricated flat‐plate structures consisting of bilayer hinges directly connected to PDMS components of arbitrary shape, and then programed the hinges to assemble the 2D precursor into a desired 3D configuration. In addition, the optically transparent PDMS plates allow for bidirectional folding when printed on both sides. As shown in **Figure** **10**a, we designed a bidirectionally folded precursor consisting of four plates connected by RGO‐TEM‐PDMS/PDMS bilayer hinges that was fabricated in a flat form (design details of the bidirectionally folded precursor are schematically given in Figure S5 in the Supporting Information). Three active hinges ≈3.5 mm in length were arranged alternately, each active hinge consists of two horizontal RGO‐TEM‐PDMS/PDMS bilayer strips, and the connected plates are transparent PDMS. Unless otherwise noted, the distance between neighboring single bilayer strips is 1 mm (in all cases). Upon uniform illumination, the adjacent plates turned toward the opposite directions (bidirectional folding), and the 2D precursors formed a triangular column with hinge angles of 90° (Figure 10a, right). Furthermore, the final triangular column structure was subsequently stretched to its original flat state repetitively a thousand times, whereupon it recovered its triangular column folded configuration spontaneously without delamination and fracture (see Figure S6 and Video S3 in the Supporting Information). This result suggests that the final folded shapes exhibit good stability and mechanical properties.

Self‐folding structures with more complex geometries also could be realized, such as boxes, pyramids, and cars. As shown in Figure 10b, a box consisting of six sides connected by bilayer hinges programed in a flat (unfolded) precursor folded itself into a rectangular box under IR light. The hinges were designed by choosing parameters from Figure 9b that resulted in a hinge angle of 90°. The entire process for folding the cube took ≈50 s, and the desired 3D shape was in good agreement with the intended shape. Moreover, when the IR light was turned off, the box permanently retained its shape. To fold a five‐sided pyramid, we created polyhedral pyramids with a square bottom face (10 × 10 mm^2^) by defining hinges (four adjacent parallel strips 4 mm in length for each hinge) with fold angles of 120°, as shown in Figure 10c. These experimental results exhibit excellent quantitative agreement with our predictions.

In addition, many other sophisticated geometries are also possible. Figure 10d illustrates a flat structure that resembles a car. The car was composed of eight active hinges and two types of hinges were used. The angles of the hinges were controlled by adjusting the length of the hinges, wherein the longer hinges were ≈4 mm long and the shorter hinges were ≈3 mm long. We then uniformly irradiated the flat precursor configuration via the IR lamp. To ensure that the final folding structure was as close as possible to the desired configuration, the initial flat template was placed on a substrate that allowed free movement. After folding to the target configuration, the folded car shown in Figure 10d (right) was obtained, and the car retained its 3D shape permanently. It is well‐known that the streamlined car shape is difficult to build from a planar precursor. Here, a sports car is constructed, as shown in Figure 10e. It can be noted from this figure that the streamlined faces are formed mainly through the bending deformation of the longer hinge components connected with the base plates. The clearance between the neighboring single hinge bilayers of the streamlined car ceiling is 2 mm wide, and the degree of the bending deformation of these longer hinge components is controlled by adjusting the irradiation time. These experimental results show that, if the bilayer hinges are properly programed, more sophisticated structures can be fabricated.

During self‐folding, the hinges undergo complex out‐of‐plane bending deformations, with associated spatially dependent variations in the curvature. The maximum strains occur at locations with the highest (unmatched) change in curvature. These locations typically remain constant throughout the folding process. Additionally, effects of fracture did not appear in any of the folded 3D devices. These results illustrate that the bilayer hinge structures exhibit excellent mechanical properties and stability.

## 3 Conclusion

In conclusion, we developed a simple and versatile approach for the preparation of light‐driven bilayer composites comprising an IR‐sensitive RGO‐TEM‐PDMS polymeric composite as an active layer and an IR‐inert PDMS as a passive layer. The RGO‐TEM‐PDMS layer can absorb IR radiation and then convert it to thermal energy to heat the TEMs, causing the TEMs to increase their volume considerably and thus induce swell. Owing to the large differences in the CTEs and Young's moduli of these two layers as a result of introducing TEMs and RGOs, the RGO‐TEM‐PDMS/PDMS bimorph platform can be driven to bend to the PDMS side under IR irradiation. The bending mechanism ascribed to the gradual expansion of the TEMs of the active layers upon heating and the thermal bilayer effect of the bilayer composites. These bilayer sheets with excellent responsive bending properties and high mechanical properties were used as active hinges for remote construction of complex 3D configurations (such as bidirectionally folded columns, boxes, pyramids, and cars) that demonstrated the three capabilities of these bilayer composites: (1) Their large deformation and excellent stability, (2) their ability to produce complex structures, and (3) their remote‐autonomous assembly upon IR irradiation. We anticipate that this polymeric composite will be useful for future studies addressing fundamental questions about the mechanics of self‐folded structures, as well as for applications in biomedical devices (e.g., flexible medical stents), space‐deployable structures (e.g., space‐deployable antennas), microfluidic devices, self‐assembling devices, and 4D printing.

## 4 Experimental Section

*Materials*: The RGOs (98% purity) with a particle size of ≈0.5–3 µm and a thickness of 0.55–3.74 nm were purchased from Chengdu Organic Chemicals Co. Ltd. of the Chinese Academy of Science. The PDMS silicone elastomer obtained from Dow Corning (Sylgard 184) was used as the host matrix. The PDMS was a two‐part solvent free flexible silicone organic polymer in the form of a base compound with a separate hydrosilane curing agent that acted as a cross‐linker. The TEMs (Clocell 120DU25) were obtained from PolyChem Alloy, and the initial microsphere diameter was determined to be 24 ± 3.2 µm by SEM (Figure S2a, Supporting Information). The TEM chosen for this study was a thermally expanding microsphere of liquid hydrocarbon surrounded by a thick thermoplastic resin shell. The thermoplastic shell softens upon heat, whereas the hydrocarbon undergoes a phase change to gas, and thus the volume of the microspheres increases considerably. The volume expansion of the microspheres is irreversible; when the heating stops, the thermoplastic shell cools down and its shape remains. All materials were used in their original form.

*Preparation of RGO‐TEM‐PDMS/PDMS Bimorph Composites*: The term “wt%” used throughout the paper refers to the ratio of additive to the PDMS base compound. The RGO‐TEM‐PDMS composites were fabricated by weighing the desired amount of RGOs and adding this to the PDMS base compound. This RGO‐PDMS base compound combination was mixed for 10 min and dispersed by sonication for 30 min to facilitate RGO distribution. Then, taking into account the additive weight, an appropriate amount of TEMs was added to the RGO‐PDMS base compound combination and further mixed for 10 min (TEMs are well‐suited for shear mixing). Next, the PDMS cross‐linker (ratio of cross‐linker to base, 1:10 by mass) was added to the RGO‐TEM‐PDMS base compound mixture and mixed for 10 min. To remove trapped air pockets, the prepared polymer was degassed for 30 min to obtain the homogeneous RGO‐TEM‐PDMS liquid polymer composites. Appropriate amounts of the RGO‐TEM‐PDMS polymer mixture were poured on 10 × 10 cm^2^ glass substrates with the tape casting process by a commercial tape casting coater (MSK‐AFA‐L800H, Hefei Kejing Materials Technology Co., Ltd.). The thickness of the obtained layer was adjusted by controlling the micrometer‐adjustable applicator (0–5000 µm). To prevent premature expansion of the incorporated expanding microspheres during the cross‐linking of the PDMS matrix, the composite was cured at the relatively low temperature of 60 °C for 1 h to fabricate the bottom RGO‐TEM‐PDMS layer (incomplete cure). Subsequently, the pristine PDMS solution was configured using the PDMS cross‐linker and base compound at a ratio of 1:10. Another PDMS layer was deposited on the incompletely cured RGO‐TEM‐PDMS layer to fabricate the bilayer structure with the same tape casting process. The bilayer composite was then postcured at 60 °C for 4 h in an oven held at vacuum. Subsequently, the RGO‐TEM‐PDMS/PDMS films were cut into strips of the desired size and peeled off of the substrate to obtain freestanding bimorph strips. In this study, the bimorph structure with a PDMS layer thickness of ≈0.7 mm and an RGO‐TEM‐PDMS/PDMS bilayer thickness of ≈1.4 mm was used in the experiment without specific mention.

*Characterization and Measurements*: The surface morphology and cross‐sectional profiles of the graphene‐based bilayer composites were characterized using a field emission scanning electron microscope (Hitachi, S‐4800) with an acceleration voltage of 5 kV. To observe the cross‐sectional profiles, the samples were prepared by immersing the films in liquid nitrogen for 10 min before fracture. Loose TEM samples were thermally expanded at different temperatures (100 or 120 °C) for 10 min, followed by SEM observations. The tensile mechanical properties of the as‐prepared composites were measured by a microforce testing system (Tytron 250, MTS System Co., USA) at 23 °C. The extension rate was 2 mm min^−1^, and the load cell was 50 N with a gauge length of ≈20 mm. The coefficient of thermal expansion of RGO‐TEM‐PDMS was measured with thermal expansion instrument (L75VS1400C, Linseis Co. Ltd., Germany) within a temperature range from room temperature to 200 °C, while the heating rate was 3 °C min^−1^. The blocking force was measured using a load cell (GSO‐10, Transducer Techniques, USA) attached to the tip end of the specimen (20 × 2 × 1.4 mm^3^) and recorded by the LabVIEW software.

*Preparation of Origami 2D Precursors with Predesigned Active Hinges at Creases*: First, the preprepared RGO‐TEM‐PDMS/PDMS bilayer strips were placed in a suitable position on a clean glass substrate according to the desired folding creases. The bilayer was patterned parallel to the folding direction of the base plates. The RGO‐TEM‐PDMS side is always convex, so the RGO‐TEM‐PDMS side of the strip was strategically placed upward or downward according to the desired folding direction. Second, the PDMS solution (ratio of base to cross‐linker, 10:1 by mass) was mixed, degassed, and poured onto the glass substrate with the tape casting process by a commercial tape casting coater. The coating thickness of the PDMS face was the same as the overall thickness of the bilayer strips. Finally, the polymeric composites were cured at 60 °C for 4 h to produce the 2D precursors, whereupon the 2D membranes were cut into the origami 2D precursors with a razor blade. Figures S4 and S5 (Supporting Information) illustrate the process of fabricating simple hinges and bidirectional‐folding 2D precursors, respectively. Other complicated origami 2D precursors also were fabricated using the same preparation method.

*Dynamic Photothermal Deformation of RGO‐TEM‐PDMS/PDMS Bimorph Composites*: To observe the deflection of the polymeric bilayers, the as‐prepared RGO‐TEM‐PDMS/PDMS bimorph sheets were cut into many strips of the same size (20 × 2 mm^2^), and an infrared lamp (250 W max, Philips BR125) was used to irradiate the bilayer strips. The photothermal bending behaviors of the RGO‐TEM‐PDMS/PDMS bimorph strips were then recorded using a digital camera (MV‐VD040SM, Microvision) with a 16 mm lens (Computar M1614‐MP2). Meanwhile, the surface temperatures of the bilayer strips during IR lamp irradiation were measured by an infrared camera (FLIR A645sc).

## Supporting information

As a service to our authors and readers, this journal provides supporting information supplied by the authors. Such materials are peer reviewed and may be re‐organized for online delivery, but are not copy‐edited or typeset. Technical support issues arising from supporting information (other than missing files) should be addressed to the authors.

Supplementary

Supplementary

Supplementary

Supplementary
