# Supplementary material for: Graphene‐Based Polymer Bilayers with Superior Light‐Driven Properties for Remote Construction of 3D Structures
Source: Adv Sci (Weinh). 2017 Jan 24;4(5):1600437. doi: 10.1002/advs.201600437 (PMC5441511; doi:10.1002/advs.201600437)
Supplement: Supplementary file 1 — Supplementary [file ADVS-4-na-s001.pdf]

Copyright WILEY-VCH Verlag GmbH & Co. KGaA, 69469 Weinheim, Germany, 2017.

ADVANCED SCIENCE Open Access

Supporting Information

for *Adv. Sci.,* DOI: 10.1002/advs.201600437

Graphene-Based Polymer Bilayers with Superior Light-Driven Properties for Remote Construction of 3D Structures

Zhenhua Tang, Ziwei Gao, Shuhai Jia, Fei Wang, and Yonglin Wang*

WILEY-VCH

Copyright WILEY-VCH Verlag GmbH & Co. KGaA, 69469 Weinheim, Germany, 2017.

Supporting Information

DOI: 10.1002/advs.201600437

Graphene-Based Polymer Bilayers with Superior Light-Driven Properties for Remote Construction of 3D Structures

Zhenhua Tang, Ziwei Gao, Shuhai Jia, Fei Wang, and Yonglin Wang*

Dr. Z. Tang, Z. Gao, Prof. S. Jia, Dr. F. Wang, Y. Wang
School of Mechanical Engineering
Xi’an Jiaotong University
Xi’an 710049, China
E-mail: shjia@mail.xjtu.edu.cn

1. Supplementary Table S1

Table S1. Young's modulus, tensile strength, and coefficient of thermal expansion (CTE) for polymer composites.

Materials	Young's modulus (MPa)	Tensile strength (MPa)	CTE (10^{-6} K^{-1})
PDMS	1.21	1.00	266–310[S1]
RGO-TEM-PDMS	1.82	1.45	1000–2000
RGO-TEM-PDMS/PDMS	1.47	0.85	NA
RGO-TEM-PDMS (at 130 °C for 3 min)	8.61	1.47	NA

This table provides some important physical parameters of some polymer composites. These experimental data are an important part of the main text by demonstrating that the active layer has the highest CTE and a larger modulus.

2. Supplementary Figures S1-S7

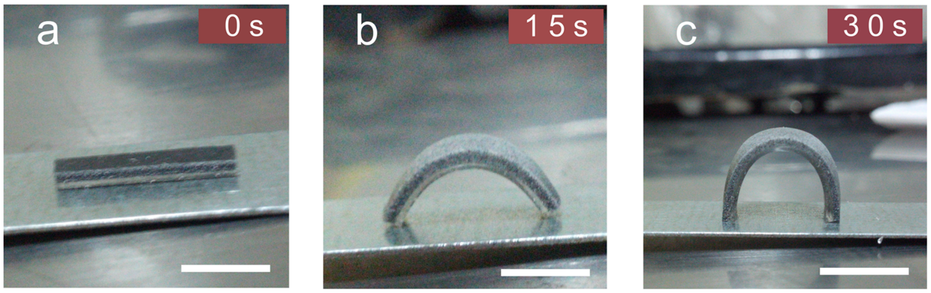

Figure S1. Optical photographs of the dynamic bending behaviors of the RGO-TEM-PDMS/PDMS bilayer sheets at different deformation process under IR irradiation. The bilayer polymeric composites always bend to PDMS side when the lamp irradiated either side. The three images correspond to (a) the initial state, (b) the state after 15 s IR irradiation, and (c) TEMs in active layer fully expanded (following 15 s IR irradiation), the specimen reached maximum deformation state. Scale bars are 10 mm.

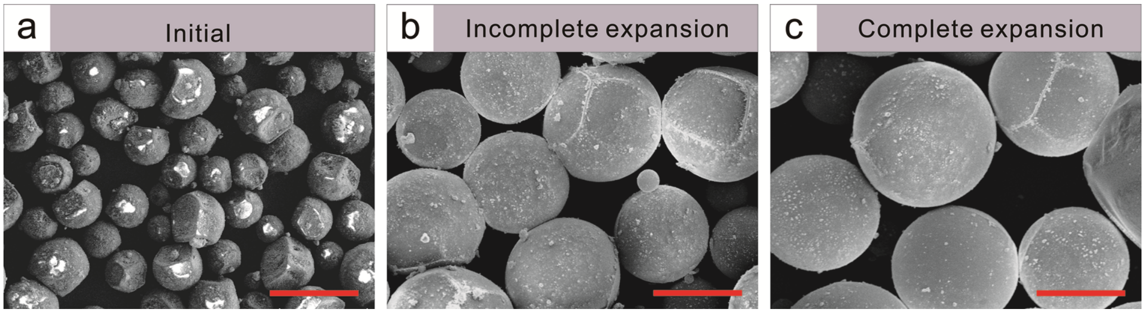

Figure S2. FE-SEM photographs of microspheres at different temperature stages. (a) FE-SEM of unexpanded microspheres (initial average diameter of 24.0 ± 3.2 µm). (b) FE-SEM of microspheres after incomplete expansion (microsphere samples at 100 °C for 10 min, average diameter of 70.3 ± 6.3 µm). (c) FE-SEM of expanded microspheres after complete expansion (microsphere samples at 120 °C for 10 min, average diameter of 86.5 ± 11.1 µm). Scale bars are 50 µm.

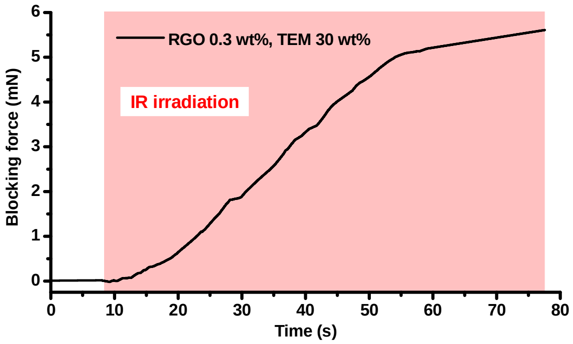

Figure S3. Blocking force of RGO-TEM-PDMS/PDMS bilayers.

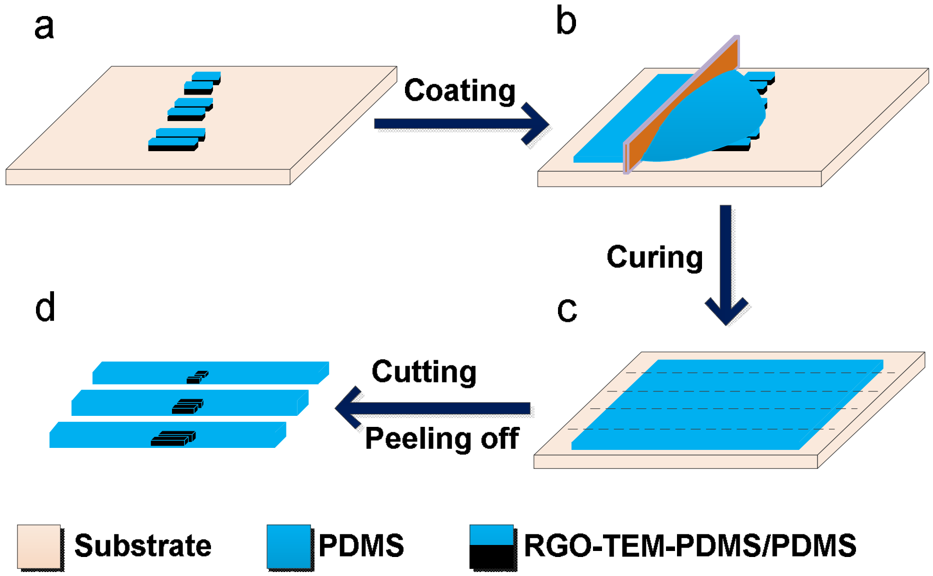

Figure S4. Fabrication scheme of polymeric bilayer hinge actuators. (a) The different lengths pre-prepared RGO-TEM-PDMS/PDMS bimorph strips (two parallel strips with 1mm clearance constituted a active hinge region) were put on the glass substrate. The fabrication process of bimorph strips of 1 mm width, 1.4 mm thickness and different lengths (1, 2, 3, 4, 5, and 6mm, respectively) are described in the Experimental Section. (b) PDMS (base compound and crosslinker at a ratio of 10:1) solution was poured on the substrate with coating process by a tape casting coater, and the coating thickness of PDMS facets equaled the thickness of the bilayer strips. (c) The polymeric composite was cured at 60 °C for 4 h to fabricate the programmable 2D precursor. (d) The film was peeled off from the glass substrate after cutting along the dash line. Finally, the hinge actuators with same dimensions (25 × 4 × 1.4 mm^3) were obtained (see Figure 5a).

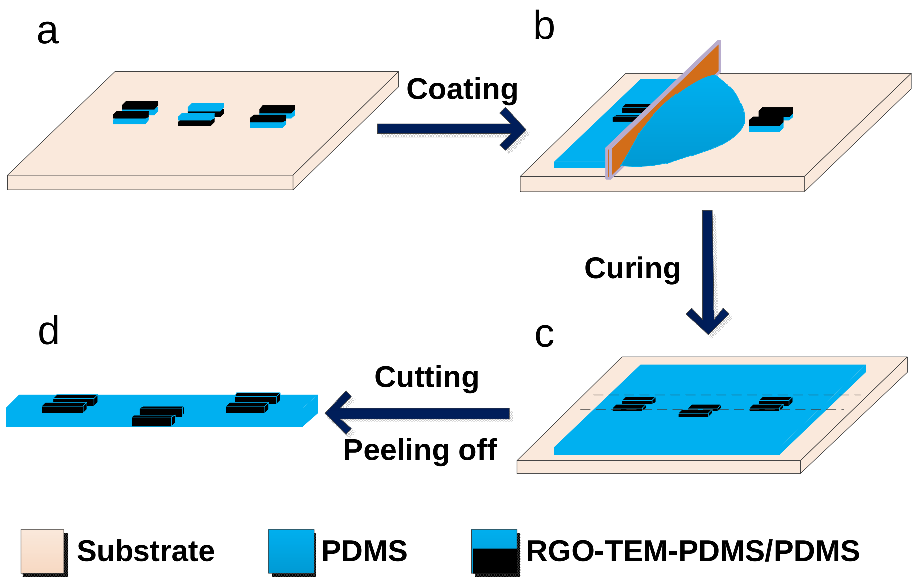

Figure S5. Fabrication scheme of triangular column 2D precursors. (a) Pattern 1.4 mm thick bilayer on substrate to form hinge region. (b) Deposit 1.4 mm thick PDMS to form 2D precursors. (c) The polymeric composite cured at 60 °C for 4 h. (d) The triangular column precursor films with dimensions 40 × 4 × 1.4 mm^3 were peeled off from the glass substrate after cutting along the dash line.

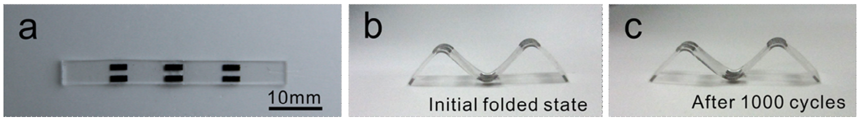

Figure S6. Cyclic testing of the origami inspired columns, under uniaxial stretching-releasing for 1000 cycles. (a) Optical image of experimental 2D precursors of the triangular columns (undeformated specimen). Optical photographs show the folded triangular columns (b) before and (c) after 1000 stretching-releasing cycles, and no delamination and fracture were observed.

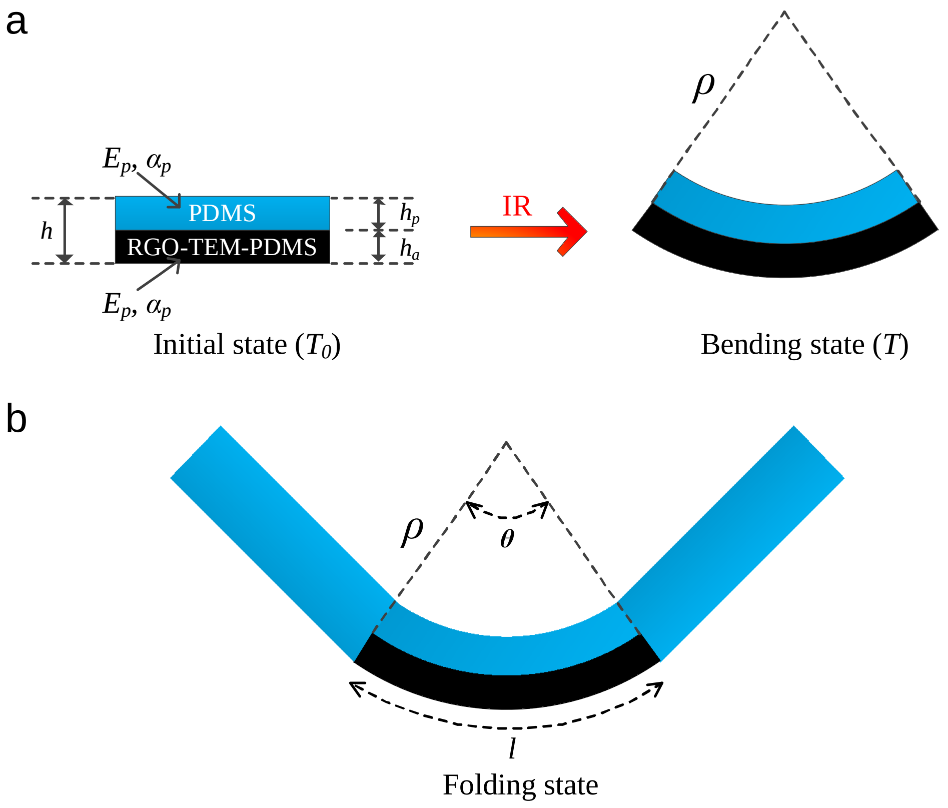

Figure S7. Definition of dimensions, radius of curvature, and bending angle for the RGO-TEM-PDMS/PDMS bilayer sheets. Figure components are not to scale. (a) Bending of the RGO-TEM-PDMS/PDMS bilayer sheet under IR irradiation. (b) Folding of the RGO-TEM-PDMS/PDMS bimorph as hinge actuation which connected two PDMS panels.

3. Model for the Bilayer Sheet

Classical beam theory by Timoshenko was first used to model bilayer bending under thermal expansion.[S2] The bilayer sheet is depicted in **Figure S7**. The sheet of total thickness h has a passive layer of PDMS with thickness h_p and an active layer of RGO-TEM-PDMS with thickness h_a so that h_p+h_a =h. The width of sheet is denoted as w. When the bilayer film is uniformly heated from temperature T_0 to T, it bends to a configuration with radius of curvature ρ due to unequal thermal expansion of the constituent layers. The model is based on five assumptions: 1) the thickness of the beam is small compared to the minimum radius of curvature; 2) the strain throughout the bilayer is determined only geometrically by the curvature; 3) there is a linear relationship between stress and strain of the material; 4)

Young's modulus and the actuation coefficient of expansion of the active layer are constant and do not depend on spatial location inside each layer; and 5) the curvature along the width of the hinge can be neglected, and deflection is only a function of length.

The relationship between the obtained radius of curvature ρ and the geometric parameters and material properties of the film was found by Timoshenko to be

$$\kappa = \frac{1}{\rho} = \frac{6\left(\alpha_a \text{-} \alpha_p\right)\left(T - T_0\right)\left(1+m\right)^2}{h\left(3\left(1+m\right)^2 + \left(1+mn\right)\left(m^2 + 1/\left(mn\right)\right)\right)}, \tag{S1}$$

where $m = h_p/h_a$ and $n = E_p/E_a$. α and E denote the thermal expansion coefficient and elastic modulus of the sheets, respectively. Subscript p and a represent the passive layer (PDMS layer) and active layer (RGO-TEM-PDMS layer), respectively. Here, the passive layer provides negligible strain compared with active layer, that is, the expanding ability of the passive layer (PDMS) α_p is assumed to be zero.[S3] Thus we use the following simple substitution

$$\varepsilon = \alpha_a\left(T - T_0\right), \tag{S2}$$

where ε is the actuation strain. Then substituting the expressions in equation S2 into equation S1, the following is obtained

$$\kappa = \frac{1}{\rho} = \frac{6\varepsilon\left(1+m\right)^2}{h\left(3\left(1+m\right)^2 + \left(1+mn\right)\left(m^2 + 1/\left(mn\right)\right)\right)}. \tag{S3}$$

The folding angle θ (°) can be calculated with the simple rules and starting from the radius of curvature as:

$$\theta = \frac{180l}{\pi\rho}, \tag{S4}$$

where l is the hinge lengths (arc length, θ). The folding angle is proportional to the hinge length l. That is, as hinge length increases, the folding angle of the hinge actuation bilayer sheet increases. The experimental data are well described by a linear relationship across the

full range of 0° to 180° (red lines, Figure 9b in main text) which matches the model trend, thus supporting the proposed approach. The folding angle was mainly controlled by the length of the bilayer hinge, this dependence allowed us to control the final shape though varying the length of the active hinges.

4. Supplementary Videos S1-S3

The strips of 20 × 2 × 1.4 mm^3 dimensions with RGO of 0.3 wt% and TEM of 30 wt% were used, and the thickness of RGO-TEM-PDMS layer and the total thickness was ~0.7 mm and ~1.4 mm, respectively.

Video S1: Bending behavior of RGO-TEM-PDMS/PDMS bilayer sheet upon IR irradiation (Philips BR125) in air. The IR light source positioned 40 mm from the PDMS side (the light incident on the PDMS side).

Video S2: Bending behavior of RGO-TEM-PDMS/PDMS bilayer sheet upon IR irradiation (Philips BR125) in air. The IR light incident on RGO-TEM-PDMS side.

Video S3: The real-time assembling process of the self-folding triangular column (Figure 10a in main text) driven by an IR light. The IR light source was positioned 180 mm from the 2D precursor.

Supplementary References

[S1] Y. Tai, G. Lubineau, Z. Yang, *Adv. Mater.* **2016**, 28, 4665.

[S2] S. Timoshenko, *J. Opt. Soc. Am.* **1925**, 11, 233.

[S3] S. Taccola, F. Greco, E. Sinibaldi, A. Mondini, B. Mazzolai, V. Mattoli, *Adv. Mater.* **2015**, 27, 1668.
